# Neocerebellar Crus I Abnormalities Associated with a Speech and Language Disorder Due to a Mutation in *FOXP2*

**DOI:** 10.1007/s12311-018-0989-3

**Published:** 2018-11-20

**Authors:** G. P. D. Argyropoulos, K. E. Watkins, E. Belton-Pagnamenta, F. Liégeois, K. S. Saleem, M. Mishkin, F. Vargha-Khadem

**Affiliations:** 1 Cognitive Neuroscience and Neuropsychiatry Section, UCL Great Ormond Street Institute of Child Health, University College London, 30 Guilford Street, London, WC1N 1EH UK; 2 Nuffield Department of Clinical Neurosciences, University of Oxford, Oxford, UK; 3 Department of Experimental Psychology, University of Oxford, Oxford, UK; 4 School of Psychology and Clinical Language Sciences, University of Reading, Reading, UK; 5 Laboratory of Neuropsychology, National Institute of Mental Health, Bethesda, MD USA; 6 Great Ormond Street Hospital for Children National Health Foundation Trust, London, UK

**Keywords:** *FOXP2*, Verbal dyspraxia, Cerebellum, Caudate nucleus, MRI, VIIa crus I

## Abstract

Bilateral volume reduction in the caudate nucleus has been established as a prominent brain abnormality associated with a *FOXP2* mutation in affected members of the ‘KE family’, who present with developmental orofacial and verbal dyspraxia in conjunction with pervasive language deficits. Despite the gene’s early and prominent expression in the cerebellum and the evidence for reciprocal cerebellum-basal ganglia connectivity, very little is known about cerebellar abnormalities in affected KE members. Using cerebellum-specific voxel-based morphometry (VBM) and volumetry, we provide converging evidence from subsets of affected KE members scanned at three time points for grey matter (GM) volume reduction bilaterally in neocerebellar lobule VIIa Crus I compared with unaffected members and unrelated controls. We also show that right Crus I volume correlates with left and total caudate nucleus volumes in affected KE members, and that right and total Crus I volumes predict the performance of affected members in non-word repetition and non-verbal orofacial praxis. Crus I also shows bilateral hypo-activation in functional MRI in the affected KE members relative to controls during non-word repetition. The association of Crus I with key aspects of the behavioural phenotype of this *FOXP2* point mutation is consistent with recent evidence of cerebellar involvement in complex motor sequencing. For the first time, specific cerebello-basal ganglia loops are implicated in the execution of complex oromotor sequences needed for human speech.

**Electronic supplementary material:**

The online version of this article (10.1007/s12311-018-0989-3) contains supplementary material, which is available to authorized users.

## Introduction

A dominantly inherited constellation of speech and language deficits in half the members of the multi-generational ‘KE family’ [1–3] has been linked to a mutation in *FOXP2* [4], the first gene to be implicated in speech and language [5]. Neural and genetic properties of this disorder may enhance our understanding of the foundations of human speech [3, 6].

Based on the neural expression pattern of the FOXP2/Foxp2 protein, Vargha-Khadem and colleagues [6] formulated a model whereby normal speech relies primarily on the modulation of activity in the ventral motor cortex via cortico-cortical pathways, as well as two major cortico-subcortical pathways, one fronto-striatal and the other fronto-cerebellar. Although abnormalities in the fronto-striatal circuit of affected KE members are well-documented [7–11], the significance of the abnormal fronto-cerebellar loops remains unexplored. The need for their detailed study is highlighted by the strikingly early and prominent expression of Foxp2/FoxP2/FOXP2 in rodent, avian and human cerebella, respectively, compared with other structures [12–19]. Moreover, the cerebellum shows massive computational power (the adult male human cerebellum contains 80% of brain neurons [20]) and an equally striking evolutionary expansion of its hemispheres [21], in concert with their cerebral association input/output areas [22, 23]. Importantly, recent evidence in mice [24] suggests that the cerebellum modulates striatal activity and cortico-striatal plasticity via a short-latency, disynaptic cerebello-striatal pathway. Likewise, the segregated, reciprocal basal ganglia-cerebellum connectivity found in non-human primates [25, 26] suggests an interplay between cortico-striatal and cortico-cerebellar circuits in motor sequence learning in the adult human brain [27, 28].

Further, neuroimaging evidence from the adult brain implicates cerebellar lobules HVI/HVIIa Crus I,^1^ left inferior frontal gyrus, premotor and supplementary motor cortex in articulatory rehearsal during verbal working memory encoding [30–33] and increased speech complexity [34]. This is in line with recent findings associating the impaired phonological working memory of affected KE members with deficits in subvocal rehearsal of speech-based material [35]. Lobules HVI/HVIIa Crus I also show somatotopically organized responses for complex movements in healthy adults [36].

In view of the above, we used advanced methods to conduct spatially precise analyses of cerebellar MRIs [37] in order to identify structural and functional abnormalities in affected KE members. We predicted that HVI/HVIIa Crus I would show the largest cerebellar structural abnormality in affected KE members relative to both unaffected members and unrelated controls. We expected that these abnormalities would be bilateral, in accordance with our previous findings on early onset speech and language disorders, where unilateral abnormality offers greater opportunity for compensation in the developing brain [38, 39]. This pattern would also be consistent with the bilateral reduction in GM volume in the caudate nucleus, and the increase in the putamen [8], as well as the bilateral HVI/HVIIa Crus I activations for verbal working memory [40] and complex movements [36]. Furthermore, HVI/HVIIa Crus I were expected to show pronounced functional abnormalities (fMRI) during non-word repetition. Problems in this task provide a reliable marker of speech and language impairment, and performance is strongly predicted by oromotor praxis in neurotypical development [41]. We also expected that the asymmetry and/or volume of HVI/HVIIa Crus I would correlate with accuracy in non-word repetition and non-verbal orofacial praxis, two key aspects of the behavioural phenotype of this mutation [42]. Finally, we examined the structural covariance of these lobules with the caudate nucleus in affected KE members, in light of the engagement of cerebellar-basal ganglia circuitry in finely timed motor control [24].

## Materials and Methods

We used structural MRI datasets acquired from different subsets of affected KE members at three time points and reported previously (time-point 1: [7–9, 43]; time-point 2: [44]; time-point 3: [11]) and fMRI data for non-word repetition [11].

### Participants

Demographic details of participants in each of the three time points are outlined in Table 1. All of the affected KE members who were available and eligible for brain imaging had been originally scanned at time point 1 (*n* = 10). At time points 2 (delay from time point 1: mean = 9.17; SD = 0.45 years) and 3 (delay from time point 1: mean = 11.50; SD = 0.58 years), subsets of those individuals (time point 2: *n* = 6; time-point 3: *n* = 4) were available for recruitment.

**Table 1:** Details of affected (‘A’), unaffected (‘U’) KE members or unrelated controls (‘C’) scanned at three time points

Participants						
Time point	Groups	*N*	Age (years)			Sex (*n* females)
			Mean	Min	Max	
1	Affected (A1–A10)	10	29.30	9	77	5
	Unaffected (U1–U5)	5	15.20	9	21	2
	Controls (C1–C9)	9	33.67	21	77	5
2	Affected (A1–A6)	6	33.00	19	57	3
	Controls* (C10–C15)	6	34.21	20	63	3
	Unaffected (U1–U4, U6–U7)	6	26.02	21	29	3
	Controls* (C16–C21)	6	27.16	23	31	3
3	Affected (A1–A4)	4	32.00	22	53	2
	Controls* (C22–C25)	4	31.75	22	52	2

*Controls were individually matched for handedness, age (± 6 years), and sex with affected/unaffected members

Time point 1 = four affected and three unaffected members were 9–18 years of age. All others were adults. No overt focal abnormalities were detectable. Unrelated controls and unaffected members had no known history of speech-language, neurological, hearing or developmental impairment. All were native English speakers

### Data Acquisition

#### MRI (Time Point 1–3)

Details of structural MRI acquisition at each time point are reported in Table S1.

#### fMRI (Time Point 3)

Further details on this study can be found in [11]. Briefly, here, two runs of 60 volumes were collected for each participant at time point 3 (see Table 1), with five task/baseline blocks (one block = 6 volumes) per run. During the task period (non-word repetition), each non-word was presented via headphones and was immediately repeated aloud by the participant. During baseline, white noise bursts were presented.

### Cerebellar Morphometry and Lobular Volumetry (Time Points 1–3)

We combined cerebellum-specific voxel-based morphometry (VBM) and lobular volumetry using SUIT (v. 3.1; http://www.icn.ucl.ac.uk/motorcontrol/imaging/suit.htm; [37]) in SPM12 (v. 6225; Wellcome Department of Cognitive Neurology, London, UK) running in Matlab 2015a (The MathWorks, Inc., Natick, MA, USA). Compared with normalization to the MNI whole-brain template, SUIT provides stronger contrast for the cerebellum, improving fissure overlap among subjects by reducing spatial variance to 1/3.

### Cerebellum-Specific VBM (Time Points 1–3)

T_1_-weighted images were re-orientated so that the origin coordinates lay over the anterior commissure and segmented into grey matter (GM), white matter (WM) and CSF, using the unified segmentation procedure [45]. The cerebellum and brainstem were isolated and a mask was created per scan, which was manually corrected using MRICron [46] (http://www.mccauslandcenter.sc.edu/mricro/mricron) as non-cerebellar regions (e.g. transverse sinus, bone marrow) are occasionally misclassified as cerebellar GM [37]. Using SUIT’s DARTEL-algorithm, the cerebellum was deformed to fit the probability maps of cortical GM and WM to an atlas template. Nonlinear deformation was applied to GM segmentation maps, which were modulated to compensate for volume changes during normalization, by multiplying the intensity value in each voxel with Jacobian determinants. The amount of GM signal in normalized images was thus preserved, with VBM statistics reflecting GM volume differences [47, 48]. Images were smoothed with an isotropic Gaussian kernel of 4 mm full-width at half-maximum (FWHM), in line with previous cerebellar VBM studies [49, 50]. GM differences between groups were assessed by voxel-wise *t* test analyses. Whenever participants in the groups were not individually matched, sex and age were entered as between-subjects covariates (ANCOVA). Comparisons were conducted separately for each subset/time point. Given the small smoothing kernels employed for spatial precision in these analyses, we applied stringent corrections for multiple comparisons (voxel peak- or cluster-level familywise error (FWE)-correction: *p* < .005) and non-stationary smoothness [51] over an individual voxel threshold of *p* < .001.

### Cerebellar Lobular Volumetry (Time Points 1–3)

The procedure involved cropping and isolating the cerebellum, SUIT normalization, re-slicing the cerebellar atlas into subject space using the deformation parameters from normalization and calculating the number of voxels in each lobule in the re-sliced images. One of the authors (GPDA) corrected the cerebellar isolation masks and the re-sliced cerebellar atlas while blind to the participant’s identity. This process resulted in volumetric measurements of cerebellar lobules (left, right I–IV, V; left, medial [vermal], or right hemispheric VI, VIIa Crus I, VIIa Crus II, VIIb, VIIIa, VIIIb, IX and X). For all comparisons, volumes were expressed both in cc and as a proportion of cerebellar cortical volume. As in VBM, ANCOVAs were used with age and sex as covariates. We predicted a main effect of group for Crus I volume, and a group **×** lobule interaction. Comparisons were conducted separately for each time-point. Significance for two-tailed tests was set at *p* < .05, using Tukey’s honestly significant difference (HSD) to correct for multiple comparisons. Violations of the sphericity assumption (tested by Mauchly’s *W*) were followed by a Huynh-Feldt correction of degrees of freedom. Hemispheric asymmetry was calculated for Crus I using the formula (right − left/right + left hemisphere) [52].

### Structure-Function Relationships (Time Point 1)

We examined the correlation (Pearson’s *r*; SPSS, v. 22) of Crus I volume and asymmetry with the behavioural measures reported to fully dissociate affected from unaffected members [42] and to correlate with the affected members’ caudate volume (time point 1; [8]): (i) *Non-word repetition*: Participants heard and repeated 40 non-words [53], half of which contained consonant clusters (‘complex’ non-words). The dependent measure was the number of complex non-words accurately repeated; (ii) *Non-verbal orofacial praxis:* Α rating scale was used to assess the performance of orofacial movements [42]. We predicted that the number of accurately repeated complex non-words and the orofacial movement ratings would correlate with Crus I volumetric measures from the same time-point.

### fMRI Analysis (Time Point 3)

After realignment, preprocessing of EPIs followed the same steps as in cerebellar VBM, apart from modulation. We compared task (non-word repetition) vs. baseline (listening to white noise bursts), using the fixed-effects analysis employed in [11], reporting regions active in the group as a whole (time point 3). We identified regions that were both activated in controls (*n* = 4; inclusive mask threshold for ‘task > baseline: *p* < .01) and less/more active in affected members (*n* = 4). The same stringent FWE corrections were applied as those in our VBM analyses.

### Structural Covariance of VIIa Crus I and Caudate Nucleus (Time Point 1)

In order to explore the structural covariance of the caudate nucleus with lobules that were structurally/functionally abnormal in affected KE family members, we first examined the correlation of the previously measured volumes of the caudate nuclei corrected for total intracranial volume (TICV) from time point 1 [8] with GM volume across the whole brain (VBM regression). The pre-processing pipeline was the same as that for cerebellar VBM, with the exception that GM images were normalized to MNI space by generating a group-specific whole-brain template (DARTEL), and an 8-mm^3^ FWHM smoothing kernel was used. Age and sex were entered as covariates and total caudate volume as a main effect regressor. We expected to identify clusters in the caudate nuclei bilaterally, given that volumetry and VBM measure the same effects [54], but also in structures the volume of which would covary with the caudate nucleus, probably due to common experience-related plasticity or mutually trophic influences, driven by environmental and genetic factors [48]. We subsequently focused on bivariate correlations (Pearson’s *r*) between TICV-corrected left/right/total caudate nucleus volumes and TICV-corrected left/right hemispheric/medial/total volumes of lobules that showed structural/functional abnormalities consistently across time-points.

## Results

Groups did not differ in TICV, whole-brain, or cerebellar cortical GM volume (between-groups comparisons across time points: *p* ≥ .10; Table 2).

**Table 2:** TICV, whole-brain GM and cerebellar cortical GM for affected, unaffected KE family members, and unrelated controls in each of the three time points; *F* and *p* values pertain to between-subjects ANOVAs

Time point	Group	TICV (cc)		Whole-brain GM (cc)		Cerebellar cortex GM (cc)	
		Mean (SD)	*F* (*p*)	Mean (SD)	*F* (*p*)	Mean (SD)	*F* (*p*)
1	Affected	1465.63 (116.49)	0.42 (0.66)	1043.30 (65.10)	1.13 (0.34)	122.62 (9.85)	2.22 (0.13)
	Unaffected	1531.15 (153.92)		1101.56 (129.95)		135.95 (13.19)	
	Controls	1504.50 (151.35)		1009.64 (135.60)		131.95 (15.31)	
2	Affected	1353.00 (84.52)	1.11 (0.35)	979.61 (67.72)	0.71 (0.51)	120.94 (11.34)	2.58 (0.10)
	Unaffected	1447.02 (157.93)		1046.18 (106.40)		134.40 (12.45)	
	Controls	1456.45 (156.60)		1032.54 (117.80)		140.90 (21.47)	
3	Affected	1479.96 (93.92)	2.64 (0.16)	1010.78 (27.92)	0.06 (0.81)	107.29 (7.76)	2.72 (0.15)
	Controls	1578.07 (75.77)		1024.14 (100.52)		119.28 (12.29)	

### Cerebellar Lobular Volumetry (Time Points 1–3)

**Fig. 1:**
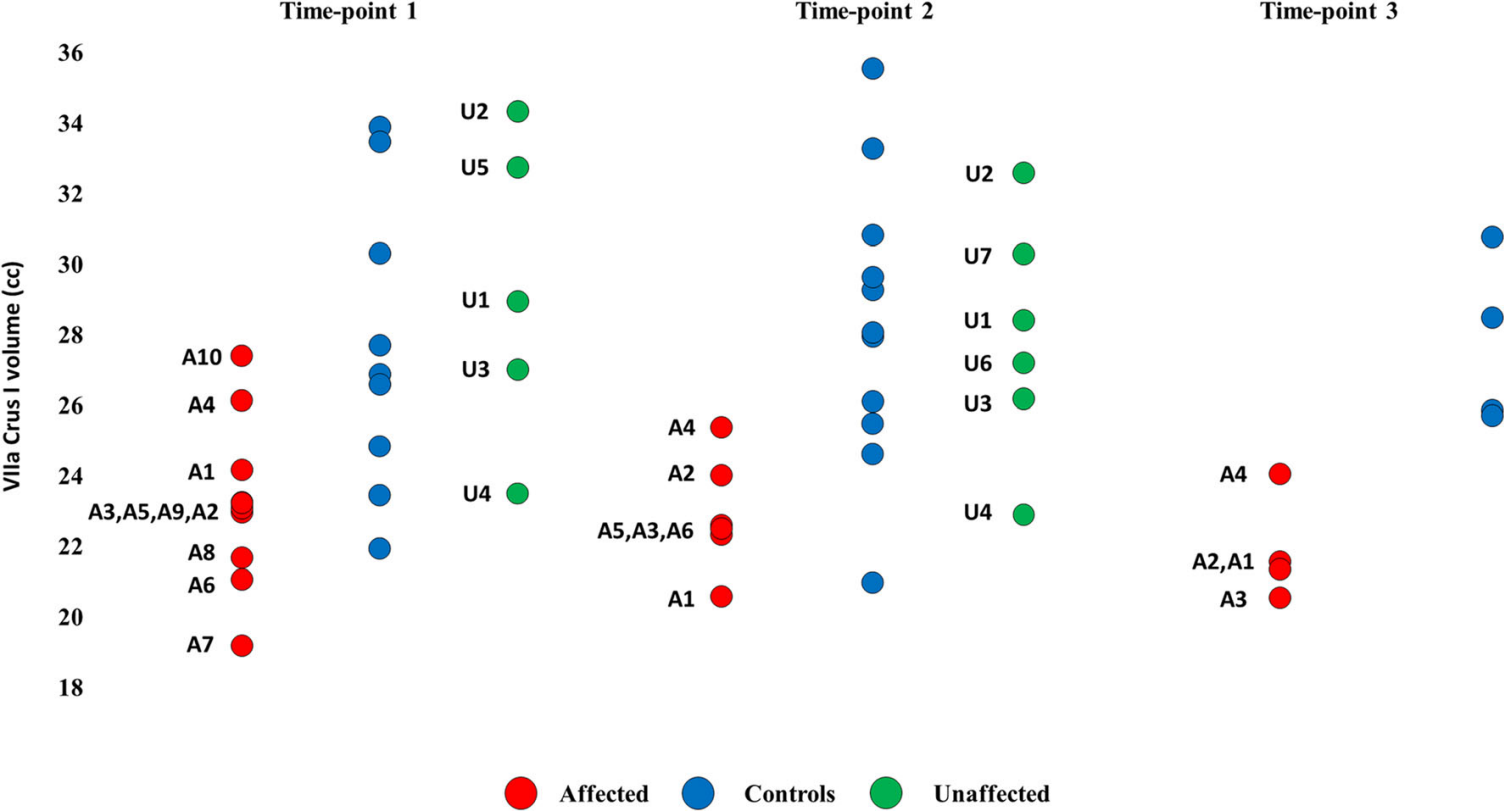
Lobular volumetry for VIIa Crus I. Red = affected. Green = unaffected. blue = controls (unrelated). Volumes of VIIa Crus I are expressed in cc. A = affected. U = unaffected KE family members

Across time points, affected members showed smaller Crus I volume relative to unaffected members and unrelated controls (Fig. 1; Table 3; time point 1 = − 18%; time point 2 = − 21%; time point 3 = − 21%), but did not differ consistently in any other lobule (Table S2). Results were replicated with (a) age and sex as between-subjects covariates (Table S3); (b) when left, right hemispheric and medial volumes were compared separately (Fig. S1); (c) when volume was expressed as percent cerebellar cortex (Table S3; Fig. S2). Unaffected KE members did not differ from unrelated controls in any lobule (time points 1 and 2).

**Table 3:** All post hoc tests were HSD-corrected for multiple comparisons; ‘affected’ = affected KE members; ‘unaffected’ = unaffected KE members; ‘controls’ = unrelated controls; the dependent measure is volume expressed in cc

Time points	Group comparisons	
1	Between-subjects ANOVAs	Group: VIIa Crus I: *F*_(2,21)_ = 6.18, *p* = .008
	(Group: affected, unaffected, controls)	(Affected vs. controls: *p* = .033; affected vs. unaffected: *p* = .014)
	Mixed-effects ANOVA	Group × lobule: *F*_(4.71, 49.41)_ = 4.03, *p* = .004
	(Group: affected, unaffected, controls; lobule: I–X)	
2	Pairwise *t* tests (affected vs. controls*)	VIIa Crus I: *t*_(5)_ = − 3.89, *p* = .012
	Repeated measures ANOVA	Group × lobule: *F*_(1.80, 9.00)_ = 9.65, *p* = .007
	(Group: affected, controls*; lobule: I–X)	
	Between-subjects ANOVA	Group: VIIa Crus I: *F*_(2,15)_ = 7.66, *p* = .005
	(Group: affected, unaffected, controls)	(Affected vs. controls: *p* = .008; affected vs. unaffected: *p* = .014)
	Mixed-effects ANOVA	Group × lobule: *F*_(4.95,37.11)_ = 4.00, *p* = .005
	(Group: affected, unaffected, controls; lobule: I–X)	
3	Paired samples *t* test (affected vs. controls*)	VIIa Crus I: *t*_(3)_ = −5.12, *p* = .014
	Repeated-measures ANOVA	Group × lobule: *F*_(5.76, 17.27)_ = 14.73, *p* = .000007
	(Group: affected, controls*; lobule: I–X)	

*Controls were individually matched for handedness, age (± 6 years), and sex with affected/unaffected members

### Cerebellum-Specific VBM (Time Points 1–3)

**Fig. 2:**
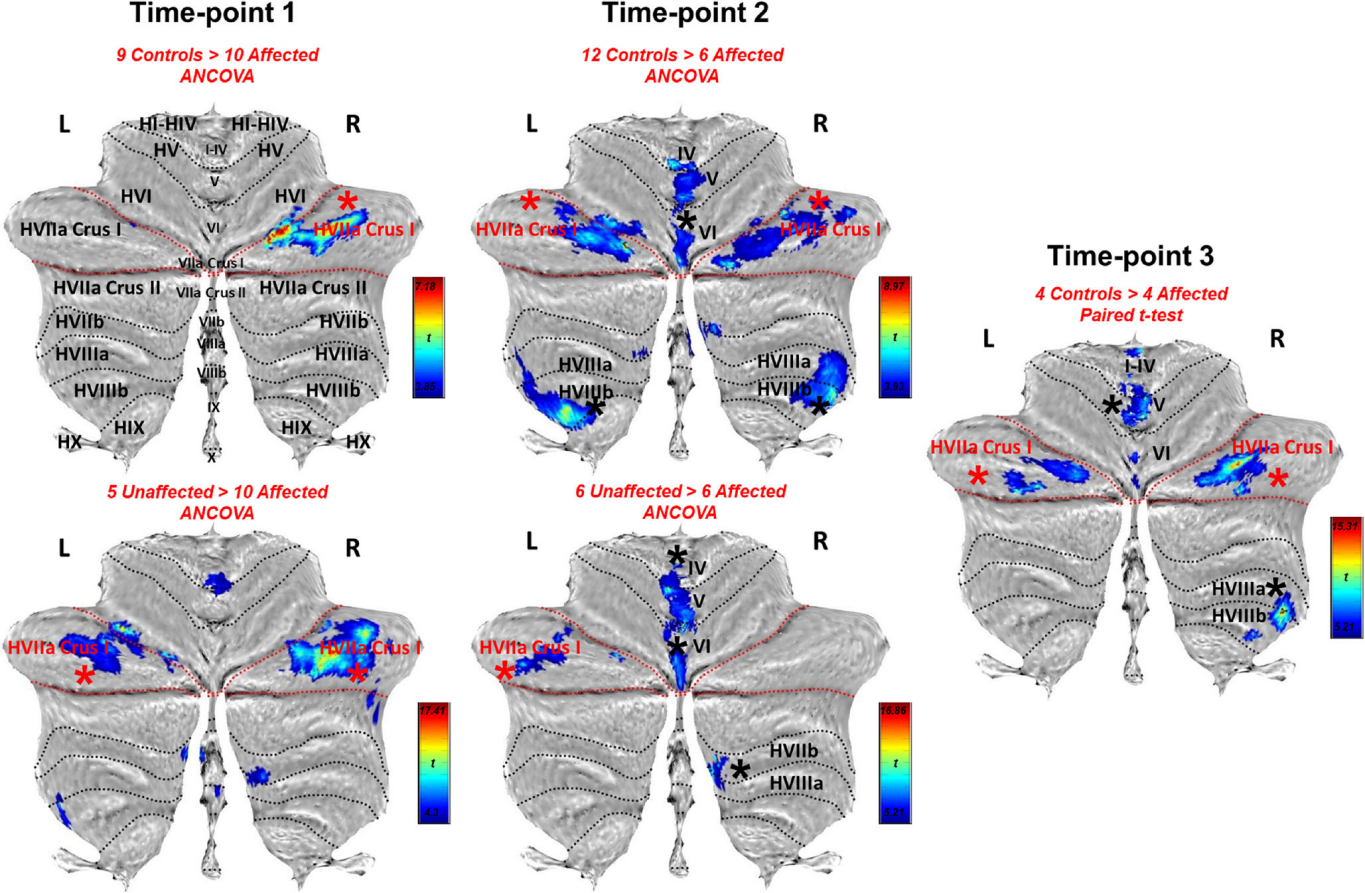
VBM. Red lines = superior-posterior and horizontal fissures, delineating VIIa Crus I in flatmap [55]; black asterisk = clusters survive correction for non-stationary smoothness and FWE (*p* < .005) over voxel threshold of *p* < .001; red asterisk = significant clusters in HVIIa Crus I are found across time points and comparisons, unlike all other lobules

The VBM analyses showed a number of regions with reduced GM volume in affected KE members relative to unrelated controls and unaffected KE members. These included medial IV–VI and hemispheric portions of VIIb–VIIIb. Nevertheless, the only discrepancy consistent across time points and comparison groups was the GM volume reduction in HVIIa Crus I of affected members (Fig. 2; Tables S4–6; Fig. S3), shown to be bilateral in most comparisons.

### Structure-Function Analysis (Tim Point 1)

**Fig. 3:**
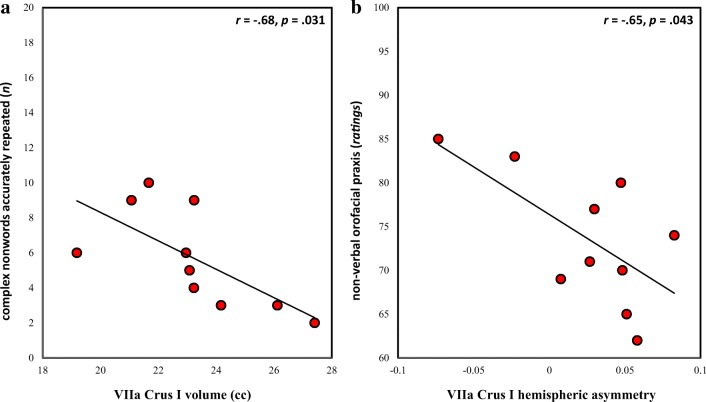
Structure-function relationships. **a** Correlation of total VIIa Crus I volume with the number of accurately repeated complex non-words. Right HVIIa Crus I volume correlated with the same measure, expressed either in cc (*r* = − .64, *p* = .048) or in percent cerebellar cortex (*r* = − .64, *p* = .047); left HVIIa Crus I volume correlated at marginal levels with the same measure when expressed in cc (*r* = − .62, *p* = .054). **b** Correlation of orofacial praxis ratings with Crus I hemispheric asymmetry; right HVIIa Crus I volume (cc) marginally correlated with the same measure (*r* = − .60, *p* = .069)

The number of accurately repeated complex non-words and the orofacial praxis ratings correlated negatively with VIIa Crus I volumetric measures (Fig. 3), but not with any other lobular volumes (all *p*s, *p* > .15). The same negative correlations have already been reported for the right and total caudate nucleus volume [8]. Using a series of partial correlation analyses, we thus sought to examine whether right/total HVIIa Crus I volumes would correlate with these scores above and beyond right/total caudate nucleus volumes, and vice versa. As expected, no significant partial correlation was shown, given the strong positive volumetric correlations of these two structures in affected KE members (Table S7).

### fMRI Analysis (Time Point 3)

**Fig. 4:**
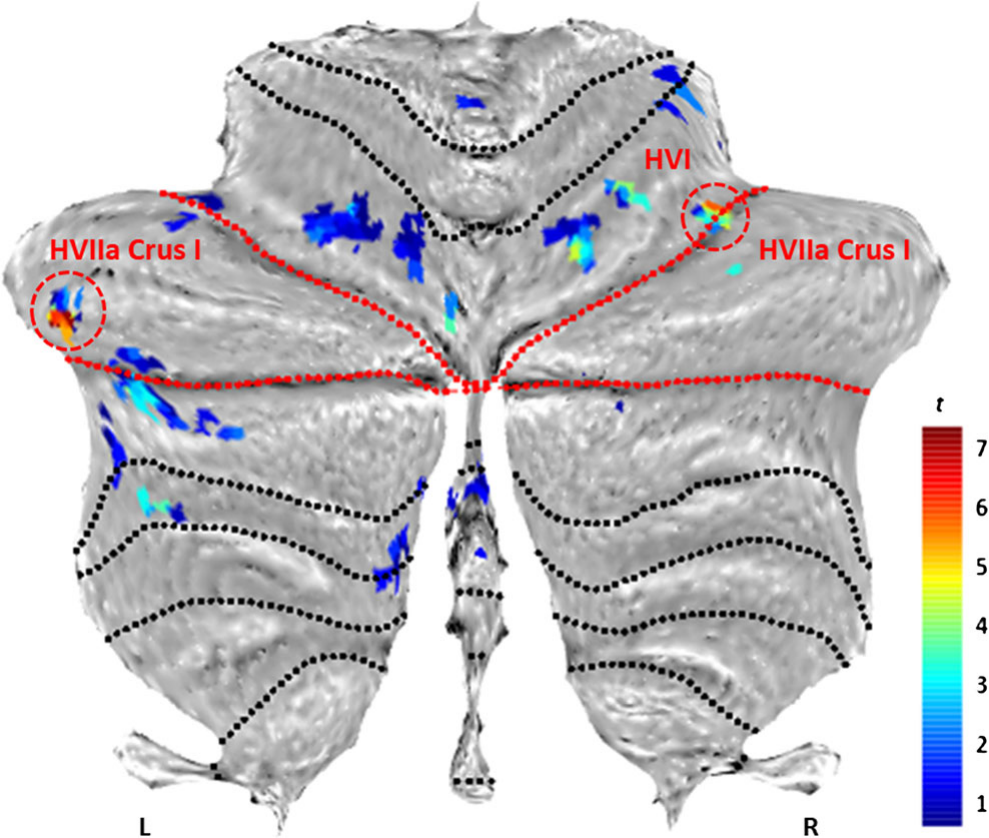
Underactivations in affected members compared to matched controls for ‘non-word repetition > noise perception’. Red lines = superior posterior and horizontal fissures, delineating VIIa Crus I in the flatmap (Diedrichsen and Zotow [55]). Red circles = clusters surviving FWE correction (*p* < .005) at peak level over *p* < .001 (unc.): left HVIIa Crus I: *x* = − 46, *y* = − 48, *z* = − 37 mm; *t* = 6.77, *z* = 6.68, *k*_*E*_ = 4 vox.; right HVI/HVIIa Crus I: *x* = 34, *y* = − 54, *z* = − 31 mm; *t* = 5.94, *z* = 5.88, *k*_*E*_ = 3 vox.). The left cluster survives a stringent inclusive threshold mask of *p* < .001. Results do not differ with a larger smoothing kernel (6-mm FWHM)

There was reduced activity in HVI/HVIIa Crus I bilaterally in affected KE members relative to matched controls during non-word repetition compared with the noise-burst baseline (Fig. 4).

### Structural Covariance of HVIIa Crus I and Caudate Nucleus (Time Point 1)

**Fig. 5:**
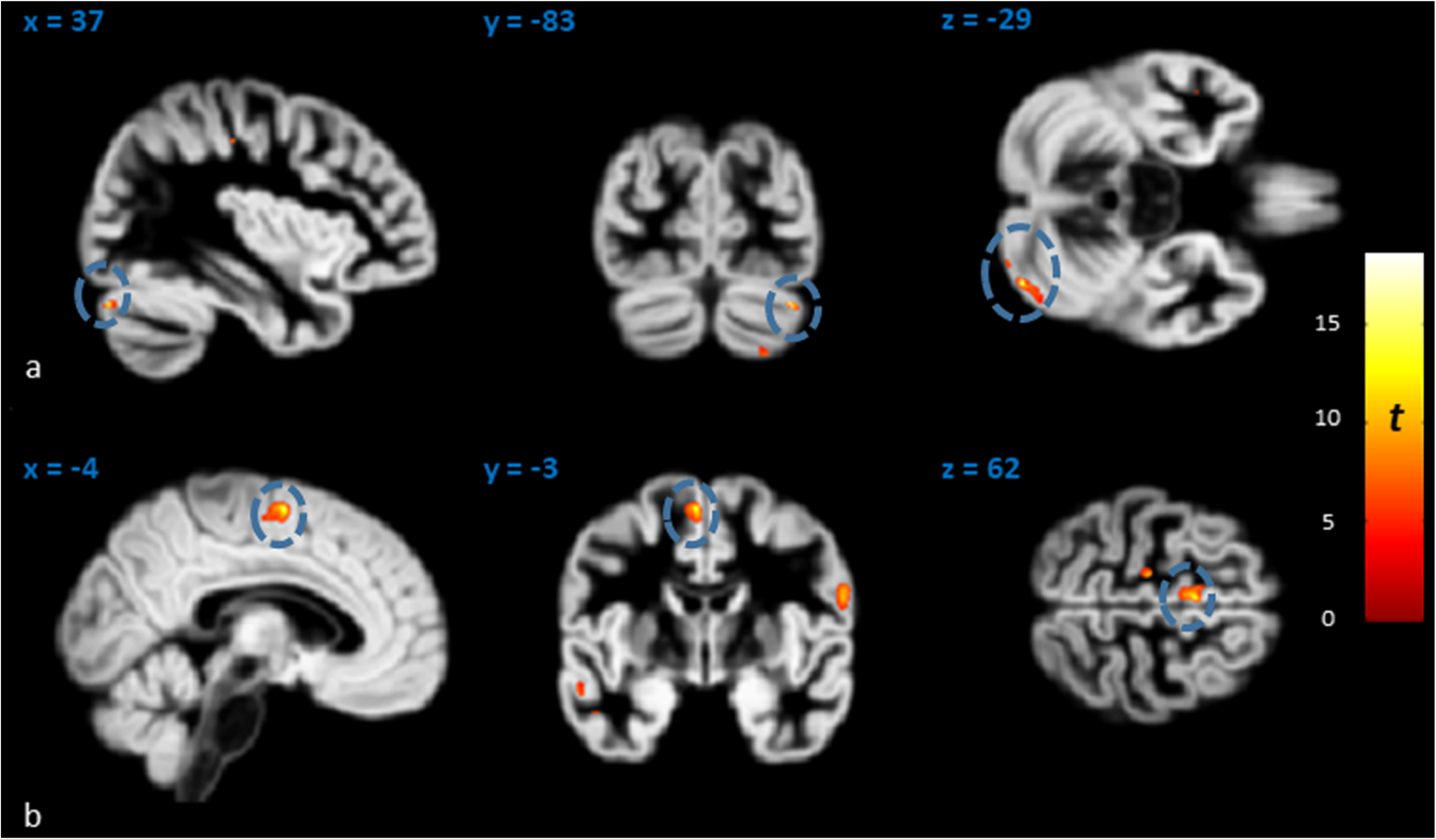
Volumes of the caudate nuclei correlated with GM volume in **a** right HVIIa Crus I and **b** left SMA. Only these two clusters (blue circles; superimposed on whole-brain GM template in MNI space) survived correction for non-stationary smoothness and FWE at cluster level (*p* < .005) over an individual voxel threshold of *p* < .001

**Fig. 6:**
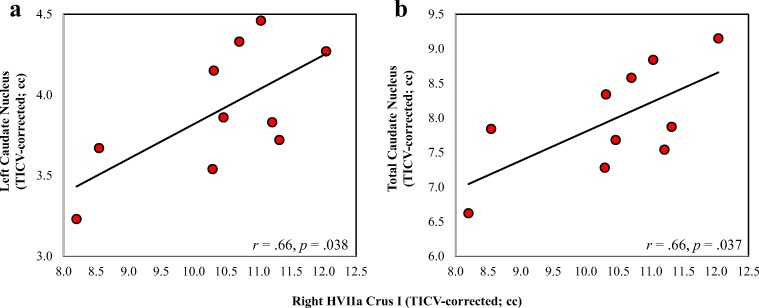
Correlation of right HVIIa Crus I volumes of affected KE family members with their **a** left caudate and **b** total caudate nucleus volumes. Total VIIa Crus I volume correlated at marginal levels with total caudate nucleus volume (*r* = .61, *p* = .060). All volumes are TICV-corrected (corrected for total intracranial volume in cc), in order to allow for the correlation of Crus I volumes calculated here with those of the caudate nuclei [8]

Crus I and caudate nuclei showed structural covariance in affected KE members. This was seen in whole-brain VBM regression, where total caudate volume expectedly correlated bilaterally with GM volume in the caudate nuclei (Fig. S4), but also with regions in the right HVIIa Crus I and the left supplementary motor area (SMA) (Fig. 5). The same relationship was seen between the previously measured volumes of the caudate nuclei [8] and those of Crus I (Fig. 6).

## Discussion

The speech and language deficits in half the members of the KE family are associated with a point mutation in *FOXP2*. Its neural and behavioural phenotype may shed light on the ontogenetic and phylogenetic foundations of articulate speech. The structural and functional abnormalities in the fronto-striatal circuitry of affected KE members have been well documented. In particular, the caudate volume reduction bilaterally represents a fundamental component of this neural phenotype that is associated with key aspects of the behavioural phenotype of this mutation. Nevertheless, very little has been known so far about the fronto-cerebellar circuits, despite the very early expression pattern of the FOXP2/Foxp2 protein in the cerebellum across species.

In this study, we identified a pronounced volume reduction (≈ 20% relative to unaffected members and unrelated controls) bilaterally in cerebellar VIIa Crus I in affected KE family members at all three of the different time points of MRI data collection. Their right hemispheric and total cerebellar Crus I volume correlated with their impaired performance in complex non-word repetition and non-verbal orofacial praxis. These two test scores reflect the core of the behavioural phenotype of this mutation [42]. Consistent with these structure-function relationships, the same lobule also showed hypoactivation bilaterally in non-word repetition. Importantly, the right hemispheric Crus I volume of affected members positively correlated with that of their left caudate nucleus, showing the same negative correlation with non-word repetition accuracy as that observed for the right (and total) caudate nucleus [8].

Our findings may thus reflect the presence of abnormalities in a cerebellar-striatal loop comprising HVIIa Crus I and the caudate nucleus. This proposal is based on evidence for reciprocal cerebellar-striatal connectivity in non-human primates [25, 26] and its role in finely timed motor control and learning in rodents [24]. It is also in line with human brain imaging studies that demonstrate resting-state functional connectivity of Crus I with the caudate nuclei [56] and the interplay of cortico-striatal with cortico-cerebellar circuits in motor sequence learning (e.g. [27, 28]. This is further supported by recent evidence for GM reduction in the caudate nucleus in patients with cerebellar atrophy [57], as well as findings highlighting the involvement of cerebellar pathology in disorders of the basal ganglia [58–60].

There are at least two possible explanations of the abnormalities described here. Firstly, Crus I regions may support speech motor sequencing across the lifespan. Studies on neurotypical adults suggest that HVI/HVIIa Crus I support motor speech sequencing [61–63], and are somatotopically organized selectively for the production of complex motor sequences [36]. While cerebellar damage is associated with ataxic dysarthria [64], the diminished sequence length effects on speech reaction times noted in cases of ataxic dysarthria have been held to reflect impaired ‘motor programming’ [65]. Interestingly, a recent study has shown effects of non-invasive stimulation of the right HVIIa Crus I/II on phonological errors in speech production (addition, deletion, transposition of phonemes) in neurotypical adults [66]. Crossed cerebellar diaschisis-related phenomena may also play an important role in apraxias of speech [67–71]. Indeed, Broca’s area (44/45), which also shows structural and functional abnormalities in affected KE members [8–11], is embedded in common functional networks with the adult HVI/HVIIa Crus I [72]. This circuitry is activated during verbal working memory encoding, motor rehearsal [30, 32–34], and modulated by difficulty in overt non-word reading [61].

A second explanation is that Crus I regions are selectively important in the pre-automatic stage of motor learning in speech acquisition. Evidence that apraxia of speech occurs as a cerebellar syndrome is quite limited [73]. There is, however, strong support for a cerebellar role in the acquisition of complex motor sequences [74–76]. With extended practice, cerebellar cortical activation decreases, paralleled by increases in cortico-striatal circuits [27], with regions close to SMA ultimately representing the automatized sequence [77]. Characteristically, the monkey pre-SMA, which, unlike SMA, is reciprocally connected with Crus I/II [78], is engaged in early motor sequence learning [79]. Similarly, pre-automatic processing of motor sequences is associated with activity in prefrontal cortex and HVI/HVIIa Crus I [80]. Regions in the caudate nucleus and Crus I are embedded within the ‘fronto-parietal control network’ [72, 81], which may be engaged during initial motor sequence learning (e.g. [82]). This dovetails with proposals that cerebellar integrity is of greater importance in earlier developmental stages [83]. Similar proposals have been made for the striatum in speech acquisition [84, 85].

Further research is required to assess these explanations. This would include larger group sizes to examine the speech-related functional abnormalities observed in Crus I for the affected KE members, given the small sample sizes analysed here. T_1_- and T_2_-weighted MRI should also be conducted at higher field strength, in order to examine structural and functional abnormalities in the dentate nucleus, to which Purkinje cells of Crus I project. Moreover, our findings do not contradict the VIIb and VIIIb volume reductions in affected KE members reported earlier [8, 9], as some contrasts here disclosed reductions in medial IV–VI and hemispheric VIIb–VIIIb. The former are the loci for the somatotopic representation of the orofacial musculature [86]; the latter may be associated with the involvement of lobule VIII in auditory [87] and somatosensory feedback processing [88]. Finally, considering the inverse correlations of non-word repetition accuracy with both Crus I and right caudate volumes, it is likely that neurodevelopmental compensatory mechanisms, functional reorganization for caudate/Crus I reduction (see discussion in [8]), and additional disruptions in synaptic pruning could be involved.

## Conclusion

Consistent with the early, homologous expression pattern of *FOXP2/Foxp2* in the human/rodent cerebellar cortical Purkinje cells, the neurodevelopmental abnormalities that we identify in lobule HVIIa Crus I of the affected members of the KE family may compromise the capacity of cerebellar-striatal circuitry to execute complex oromotor sequences. Our findings thus point towards abnormality in a circuit linking cerebellar lobule HVIIa Crus I with the caudate nucleus. This loop may be a fundamental component of the neural apparatus that enables the finely timed coordination of complex oromotor sequences needed for human speech. Research on the neurobiology of speech and language acquisition and processing needs to examine the complex interplay between cortico-striatal and cortico-cerebellar circuits, rather than their contributions in isolation from each other.

## Electronic supplementary material

ESM 1 (DOC 934 kb)

## Footnotes

1 We follow the nomenclature used in the ‘MRI Atlas of the Human Cerebellum’ [29]: cerebellar lobules are labelled ‘I-X’ from the anterior-superior border, through posterior-superior, posterior-inferior, to the anterior-inferior border. The nomenclature is based on that of Larsell and Jansen [21], where cerebellar hemispheres are distinguished from the vermis with the ‘H’ prefix. The atlas of Schmahmann and colleagues [29] uses this prefix to refer specifically to the hemispheres, and the adjective ‘vermal’ to refer to vermal compartments of a lobule. No prefix is used when referring to the entire lobule.
