# Supplementary material for: Neocerebellar Crus I Abnormalities Associated with a Speech and Language Disorder Due to a Mutation in FOXP2
Source: Cerebellum. 2018 Nov 20;18(3):309–19. doi: 10.1007/s12311-018-0989-3 (PMC6517346; doi:10.1007/s12311-018-0989-3)
Supplement: Supplementary file 1 — (DOC 934 kb) [file 12311_2018_989_MOESM1_ESM.doc]

**Electronic Supplementary Material**

| **TIME-POINT** | **Structural MRI** | | | | | | | | |
| --- | --- | --- | --- | --- | --- | --- | --- | --- | --- |
|  | **Scanner** | | **Sequence** | **TR** | **TE** | **Flip Angle** | **FoV** | **Matrix size** | **Voxel size** |
|  |  |  |  | **(ms)** | | **(º)** | **(mm)** | | |
|  | **1** | 1.5T Siemens SP | MPRAGE | 10 | 4 | 12 | 250 | 256 × 256 × 128 | 0.98 × 0.98 × 1.25 |
|  | **2** | 1.5T Siemens Vision | FLASH | 16.8 | 5.7 | 21 | 200 | 256 × 256 × 160 | 0.78 × 0.78 × 1.00 |
|  | **3** | 1.5T Siemens Vision | FLASH | 31 | 11 | 40 | 192 | 224 × 256 × 176 | 1.00 × 1.00 × 1.00 |

**Table S1:** Details of structural T1-weighted MRI acquisition in each of the three time-points.


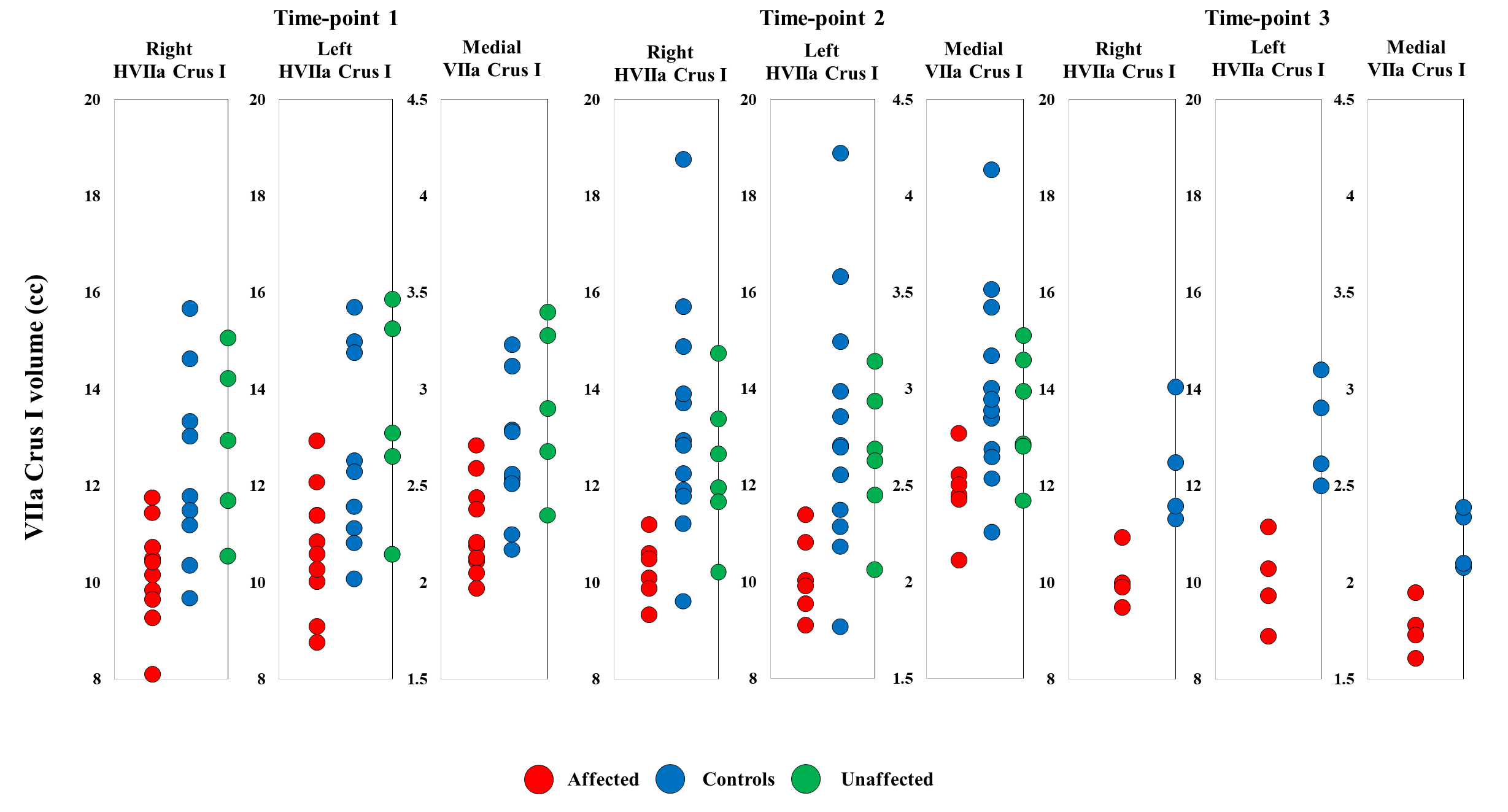


**Fig. S1:** Lobular volumetry for right, left hemispheric, and medial / vermal compartments of lobule VIIa Crus I. Red: Affected; Green: Unaffected; Blue: Controls (unrelated); Volumes of VIIa Crus I are expressed in cc.

|  | **Cerebellar Lobular Volumetry** | | |
| --- | --- | --- | --- |
| **TIME-POINT** | **1** | Between-subjects ANOVAs  (Group: affected, unaffected, controls) | **Group: I-IV:** *F* = 8.88, *p* = .002 |
|  | **2** | Pair-wise *t*-tests  (affected vs. controls*) | **I-IV**: *t* = -2.70, *p* = .043; **V**: *t* = -3.58, *p* = .016 |
|  |  | Between-subjects ANOVA  (Group: affected, unaffected, controls) | **Group: V**: *F* = 3.94, *p* = .042 |
|  | **3** | Paired samples *t*-test  (affected vs. controls*) | **I-IV**: t = -8.90, p = .003; **V**: t = -13.62, p = .001; **VI**: t = -4.14, p = .026 |

**Table S2:** Lobules other than VIIa Crus I in which affected KE members showed reduced volumes: ‘affected’: affected KE members; ‘unaffected’: unaffected KE members; ‘controls’: unrelated controls; I-X: cerebellar lobules. *ns*: *p* > .05; dependent measure: volume (cc); *: controls were individually matched for handedness, age (+/- 6 years), and sex with affected / unaffected members.


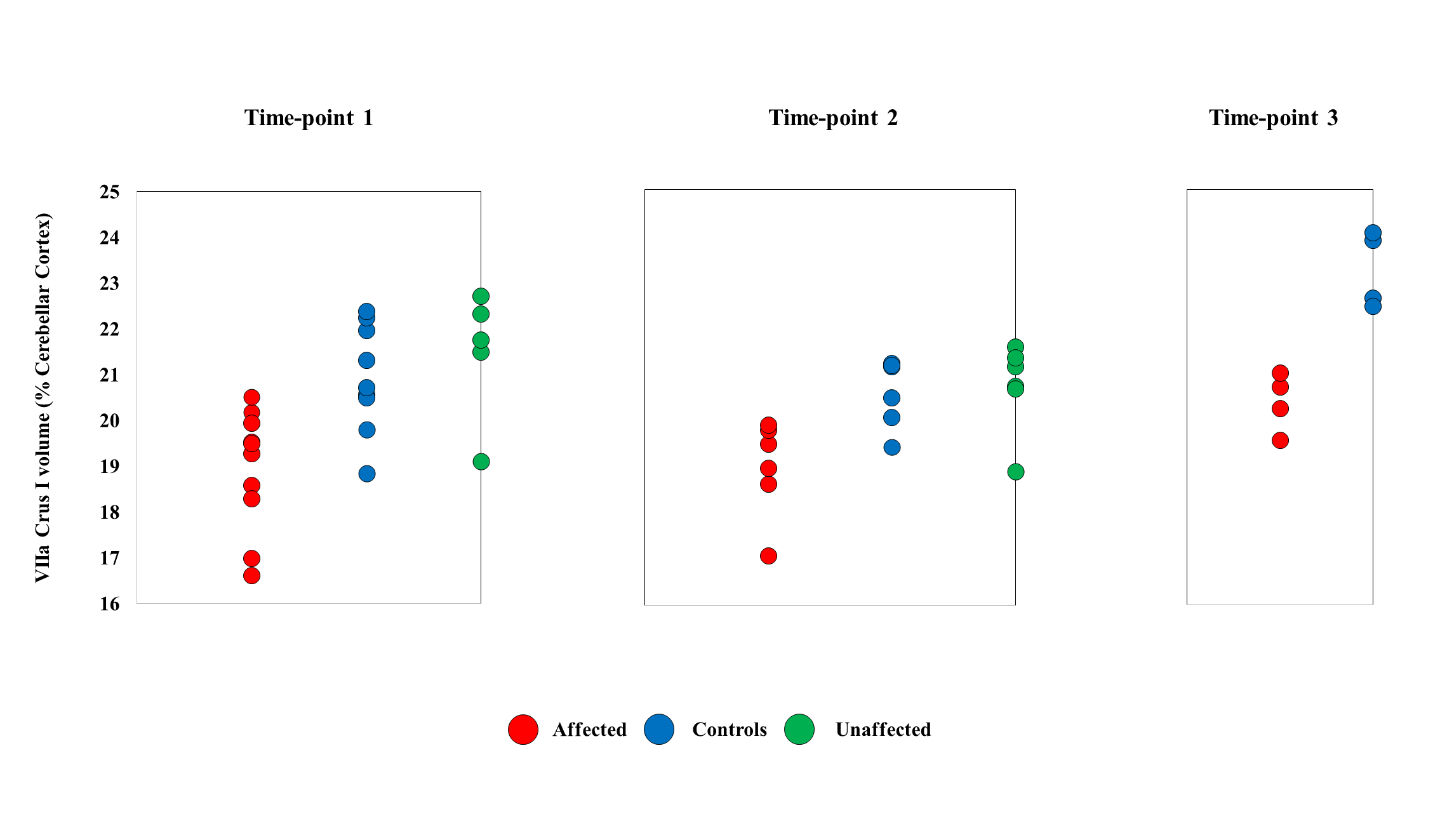
**Fig. S2:** VIIa Crus I volumetry. Red: Affected; Green: Unaffected; Blue: Controls (unrelated); Volumes expressed as % total cerebellar cortex.

| **Cerebellar Lobular Volumetry** | | | |
| --- | --- | --- | --- |
|  | **Comparison** | **Dependent measure = cc** | **Dependent measure = % cerebellar cortex** |
| **TIME-POINT 1** | Between-subjects ANOVAs (Group: affected, unaffected, controls) | **Group: VIIa Crus I:** *F*_(2,21)_ = 6.18, *p* = .008 (affected vs. controls: *p* = .033; affected vs. unaffected: *p* = .014; controls vs. unaffected: *ns*)  **Other lobules:** I-IV: *F*_(2,21)_ = 8.88, *p* = .002 | **Group: VIIa Crus I:** *F*_(2,21)_ = 8.70, *p* = .002 (affected vs. controls: *p* = .008; affected vs. unaffected: *p* = .005; controls vs. unaffected: *ns*) **Other lobules:** IX: *F*_(2,21)_ = 6.75, *p* = .005 |
|  | Mixed-effects ANOVA (Group: affected, unaffected, controls; Lobule: I-X) | **Group × Lobule:** *F*_(4.71, 49.41)_ = 4.03, *p* = .004 | **Group × Lobule:** *F*_(7.52, 78.94)_ = 3.84, *p* = .001 |
| **TIME-POINT 2** | Pair-wise *t*-tests (affected vs. controls*) | **VIIa Crus I:** *t* = -3.89, *p* = .012  **Other lobules:** I-IV: *t* = -2.70, *p* = .043; V: *t* = -3.58, *p* = .016 | **VIIa Crus I:** *t* = -5.91, *p* = .002  **Other lobules:** X: *t* = 2.68, *p* = .044 |
|  | Repeated measures ANOVA (Group: affected, controls*; Lobule: I-X) | **Group × Lobule:** *F*_(1.80, 9.00)_ = 9.65, *p* = .007 | **Group × Lobule:** *F*_(8.24, 41.21)_ = 7.43, *p* = .000004 |
|  | Pair-wise *t*-tests (unaffected vs. controls*) | **All lobules:** *ns* | V: *t* = 3.21, *p* = .024; I-IV: *t* = 2.07, *p* = .03 |
|  | Repeated measures ANOVA (Group: unaffected, controls*; Lobule: I-X) | **Group × Lobule:** *ns* | **Group × Lobule:** *ns* |
|  | Between-subjects ANOVA (Group: affected, unaffected, controls) | **Group: VIIa Crus I:** *F*_(2,15)_ = 7.66, *p* = .005 (affected vs. controls: *p* = .008; affected vs. unaffected: *p* = .014; controls vs. unaffected: *ns*)  **Other lobules:** V: *F*_(2,15)_ = 3.94, *p* = .042 | **Group: VIIa Crus I:** *F*_(2, 15)_ = 6.65, *p* = .009 (affected vs. controls: *p* = .022; affected vs. unaffected: *p* = .013; controls vs unaffected: *ns*)  **Other lobules:** *ns* |
|  | Mixed-effects ANOVA (Group: affected, unaffected, controls; Lobule: I-X) | **Group × Lobule:** *F*_(4.95,37.11)_ = 4.00, *p* = .005 | **Group × Lobule:** *F*_(6.08, 45.62)_ = 2.33, *p* = .047 |
| **TIME-POINT 3** | Paired samples *t*-test  (affected vs. controls*) | **VIIa Crus I:** *t* = -5.12, *p* = .014  **Other lobules:** I-IV: *t* = -8.90, *p* = .003; V: *t* = -13.62, *p* = .001; VI: *t* = -4.14, *p* = .026 | **VIIa Crus I:** *t* = -13.04, *p* = .001  **Other lobules:** *ns* |
|  | Repeated-measures ANOVA (Group: affected, controls*; Lobule: I-X) | **Group × Lobule:** *F*_(5.76, 17.27)_ = 14.73, *p* = .000007 | **Group × Lobule:** *F*_(6.03, 18.09)_ = 11.02, *p* = .00003 |

**Table S3:** Cerebellar lobular volumetry; volumes expressed as cc and % cerebellar cortex; post-hoc tests were HSD-corrected for multiple comparisons; ‘affected’: affected KE members; ‘unaffected’: unaffected KE members; ‘controls’: unrelated controls; I-X: cerebellar lobules. *ns*: *p* > .05; *: controls were individually matched for handedness, age (+/- 6 years), and sex with affected / unaffected members.

| CEREBELLAR VBM | | | | | | | | | |
| --- | --- | --- | --- | --- | --- | --- | --- | --- | --- |
| TIME-POINT 1 | **Comparison** |  | **Cluster** | **Peak** | | | | | |
|  |  | **ANCOVA** | ***k_E_*** | ***t*** | *z* | **x _mm_** | **y**  **_mm_** | **z**  **_mm_** | **Lobule** |
|  | **5 Unaffected > 10 Affected** |  | **2735** | **17.41** | **5.54** | **45** | **-59** | **-33** | **r-HVIIa Crus I** |
|  |  |  | **273** | **13.69** | **5.16** | **-27** | **-63** | **-28** | **l-HVI / HVIIa Crus I** |
|  |  |  | **704** | **7.72** | **4.18** | **-36** | **-68** | **-25** | **l-HVIIa Crus I** |
|  | **10 Affected > 5 Unaffected** |  | *ns* (FWE-corr, *p* ≥.14) | | | | | | |
|  | **9 Controls > 10 Affected** |  | **443** | **7.18** | **4.49** | **19** | **-67** | **-32** | **r-HVI / HVIIa Crus I** |
|  |  |  | **1471** | **6.93** | **4.41** | **40** | **-71** | **-27** | **r-HVIIa Crus I** |
|  | **10 Affected > 9 Controls** |  | 468 | 7.67 | 4.64 | 11 | -36 | -21 | r-I-IV |
|  |  |  | 598 | 7.56 | 4.60 | -11 | -49 | -43 | l-HIX / HVIIIb |
|  |  |  | 401 | 7.31 | 4.53 | 13 | -45 | -43 | r-HIX |
|  | **5 Unaffected > 9 Controls** |  | 347 | 8.54 | 4.20 | -22 | -79 | -38 | l-HVIIa Crus II / I |
|  |  |  | 350 | 8.17 | 4.12 | 0 | -75 | -28 | m-VI / VIIa Crus I |
|  |  |  | 351 | 8.75 | 4.24 | 18 | -87 | -36 | r-HVIIa Crus II |
|  | **9 Controls > 5 Unaffected** |  | *ns* (FWE-corr, *p* ≥.2) | | | | | | |
| TIME-POINT 2 | **6 Controls* > 6 Affected** | **paired t-test** | **535** | **16.12** | **4.30** | **-22** | **-69** | **-28** | **l-HVI / HVIIa Crus I** |
|  |  |  | 814 | 28.84 | 4.90 | 26 | -50 | -51 | r-HVIIIb / HVIIIb |
|  | **6 Affected > 6 Controls*** |  | 49 | 16.47 | 4.33 | 34 | -79 | -42 | r-HVIIa Crus II |
|  | **6 Unaffected > 6 Controls*** |  | *ns* (FWE-corr, *p* ≥.055) | | | | | | |
|  | **6 Controls > 6 Unaffected** |  | *ns* (FWE-corr, *p* ≥.036) | | | | | | |
|  | **12 Controls > 6 Affected** | **ANCOVA** | **1215** | **8.97** | **4.86** | **-23** | **-80** | **-25** | **l-HVIIa Crus I / HVI** |
|  |  |  | **1555** | **7.91** | **4.60** | **20** | **-88** | **-26** | **r-HVIIa Crus I / HVI** |
|  |  |  | 786 | 8.38 | 4.72 | -18 | -43 | -54 | l-HVIIIb,a |
|  |  |  | 1012 | 7.54 | 4.50 | 29 | -39 | -48 | r-HVIIIb,a |
|  |  |  | 315 | 6.42 | 4.15 | 0 | -76 | -25 | medial VI |
|  | **6 Affected > 12 Controls** |  | *ns* (FWE-corr, *p* ≥.012) | | | | | | |
|  | **6 Unaffected > 6 Affected** |  | **268** | **8.92** | **3.87** | **-51** | **-65** | **-36** | **l-HVIIa Crus I** |
|  |  |  | 713 | 16.86 | 4.69 | -5 | -57 | -7 | l-HV / r-HV / l-I-IV |
|  |  |  | 127 | 15.78 | 4.61 | 11 | -74 | -45 | r-HVIIb / HVIIIa |
|  |  |  | 294 | 9.69 | 3.98 | -1 | -73 | -22 | m-VI |
|  | **6 Affected > 6 Unaffected** |  | *ns* (FWE-corr, *p* ≥.063) | | | | | | |
| TIME-POINT 3 | **4 Controls* > 4 Affected** | **paired t-test** | **1115** | **15.31** | **4.57** | **37** | **-70** | **-32** | **r-HVIIa Crus I** |
|  |  |  | 265 | 9.75 | 3.99 | 2 | -58 | -17 | r-HV / l-HV / r-I-IV / l-I-IV |
|  |  |  | 210 | 14.36 | 4.49 | 28 | -43 | -47 | r-HVIIIb |
|  | **4 Affected > 4 Controls*** |  | 390 | 10.27 | 4.06 | -35 | -41 | -37 | l-HVI/HV |

**Table S4:** Cerebellum-specific VBM; clusters are corrected for non-stationary smoothness and FWE (*p* < .005) at cluster level over an individual voxel threshold of *p* < .001; r: right; l: left; m: medial / vermal; H: hemispheric; ANCOVA: age and sex were added as between-subjects covariates; *k_E_:* cluster size (number of voxels); ‘unaffected’: unaffected ΚΕ members; ‘controls’: unrelated controls; *: controls were individually matched for handedness, age (+/- 6 years), and sex with affected / unaffected members.

Tables S5-6, Fig. S3: As there were 4 affected members common across time-points (table 1), we investigated whether the same abnormalities would be found in the other 6 affected members that had participated at time-point 1. We thus compared these 6 members against unrelated controls, unaffected members, and the 4 affected members common across time-points. The group of 6 affected members did not differ in Crus I volume reduction from the other 4, showing the same reduction relative to unrelated controls and unaffected members.

| **Cerebellar VBM** | | | | | | | |
| --- | --- | --- | --- | --- | --- | --- | --- |
| **Comparison** | **Cluster** | **Peak** | | | | | Lobule |
|  | *k_E_* | *t* | *z* | x | y | z |  |
| **Unaffected > A5-A10** | 116 | 30.96 | 4.97 | 36 | -70 | -44 | r HVIIa Crus II / I |
|  | 480 | 18.59 | 4.46 | 38 | -70 | -33 | r HVIIa Crus I |
|  | 141 | 13.14 | 4.08 | 48 | -52 | -47 | r HVIIa Crus II / I |
|  | 43 | 14.85 | 4.21 | -44 | -49 | -43 | l HVIIa Crus II |
|  | **39** | **16.68** | **4.34** | **29** | **-73** | **-26** | **r HVIIa Crus I** |
|  | 100 | 10.67 | 3.84 | 14 | -81 | -32 | r HVIIa Crus II |
|  | **127** | **9.16** | **3.65** | **-49** | **-60** | **-40** | **l HVIIa Crus I** |
|  | 89 | 19.4 | 4.5 | -28 | -32 | -31 | l HV |
| **A5-A10 > Unaffected** | 87 | 15.15 | 4.24 | -18 | -55 | -14 | l HVI |
| **Controls > A5-A10** | 645 | 9.79 | 4.6 | 47 | -67 | -25 | r HVIIa Crus I |
| **A5-A10 > Controls** | *ns* (FWE *p* > .028) | | | | | | |
| **A1-A4 > A5-A10** | 127 | 26.22 | 4.37 | -27 | -37 | -31 | l HV-HVI |
|  | 21 | 22.19 | 4.22 | 10 | -66 | -55 | r HVIIIa – HVIIIb |
| **A5-A10 > A1-A4** | *ns* (FWE *p* > .58) | | | | | | |

**Table S5:** Comparison of affected ΚΕ members A5-A10 against unrelated controls and unaffected ΚΕ members (time-point 1) with VBM; clusters corrected for non-stationary smoothness and FWE (*p* < .005) over voxel threshold of *p* < .001; r: right; l: left; m: medial / vermal; H: hemispheric; age and sex added as covariates; *k_E_:* cluster size.

**
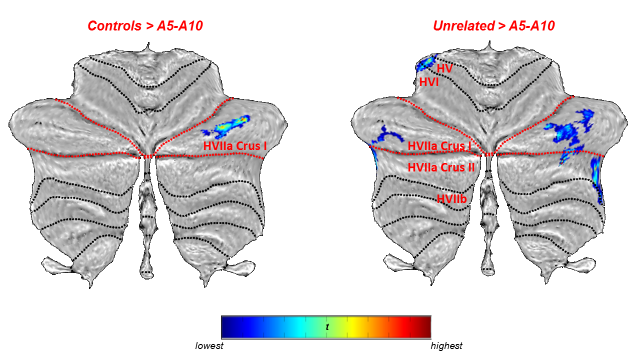
Fig. S3:** Comparison of affected ΚΕ members A5-A10 against unrelated controls and unaffected ΚΕ members (time-point 1). Clusters depicted survive correction for non-stationary smoothness and FWE (p < .005) over an individual voxel threshold of p < .001.

| **Cerebellar Lobular Volumetry** | | |
| --- | --- | --- |
| Comparison | **Dependent measure = cc** | **Dependent measure = % cerebellar cortex** |
| **Between-subjects ANOVAs (Group: A5-A10, unaffected, controls)** | **Group: VIIa Crus I:** *F*_(2,17)_ = 4.75, *p* = .023; affected vs. controls: *p* = .059; affected vs. unaffected: *p* = .028; controls vs. unaffected: *ns*  **Other lobules: I-IV:** *F*_(2,17)_ = 5.15, *p* = .023 | **Group: VIIa Crus I:** *F*_(2,17)_ = 8.85, *p* = .002; affected vs. control: *p* = .007; affected vs. unaffected: *p* = .004; controls vs. unaffected: *ns*  **Other lobules: IX:** *F*_(2,17)_ = 5.23, *p* = .017; **X:** *F*_(2,17)_ = 4.95, *p* = .02 |
| **Mixed-effects ANOVA (Group: A5-A10, unaffected, controls; Lobule: I-X)** | **Group × Lobule:** *F*_(4.85,41.18)_ = 3.35, *p* = .013 | **Group × Lobule:** *F*_(8.16, 69.37)_ = 3.63, *p* = .001 |
| **Independent samples t-test (A1-A4 vs. A5-A10)** | *ns* | **X:** *t* = 3.09, *p* = .015 |

**Table S6:** Comparison of affected ΚΕ members A5-A10 against unrelated controls and unaffected ΚΕ members (time-point 1) on cerebellar lobular volumes; *ns*: non-significant, i.e. p > .05.

**
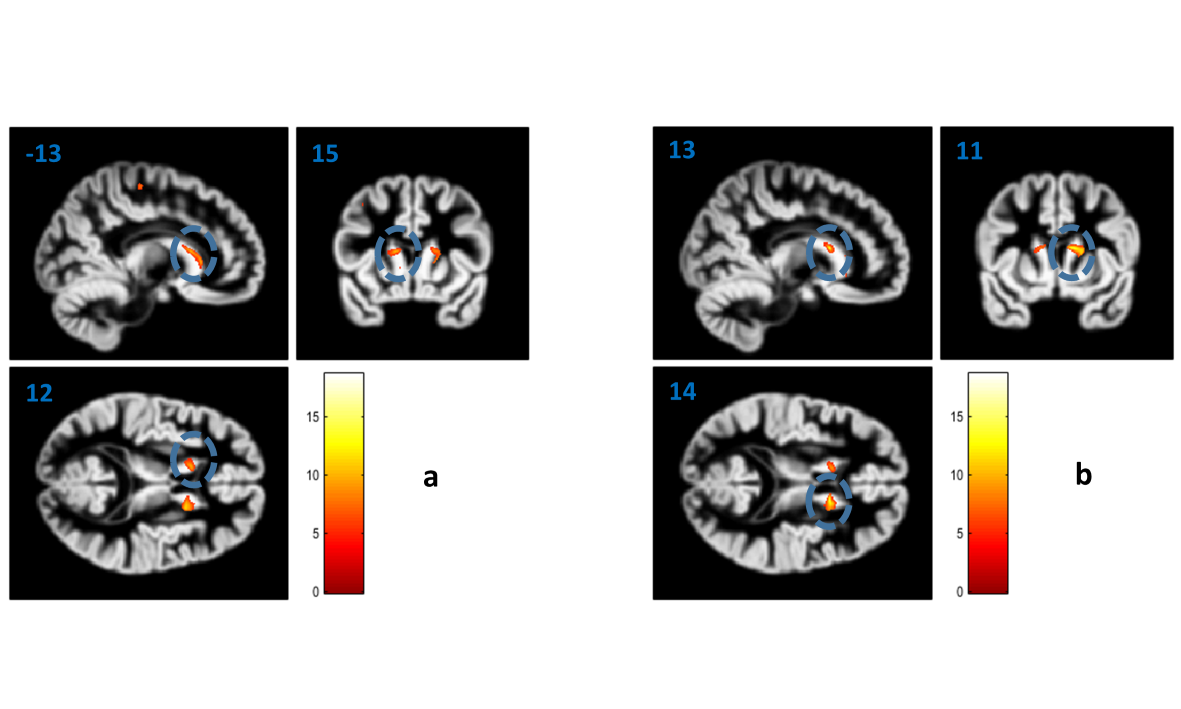
Fig. S4:** Expectedly, caudate volume correlated with GM volume in a: left caudate head/body; b: right caudate head/body; clusters marked with a circle survive correction for non-stationary smoothness and FWE (p < .005) over an individual voxel threshold of p < .001.

| **Partial Correlations with Complex Non-Word Repetition** | | | |
| --- | --- | --- | --- |
| **Control Variable (TICV-corrected Volume)** | **Independent Variable (TICV-corrected Volume)** | **r** | **p (two-tailed)** |
| Total HVIIa Crus I | Total Caudate Nucleus | -.44 | .23 |
| Total Caudate Nucleus | Total HVIIa Crus I | -.46 | .21 |
| Right HVIIa Crus I | Total Caudate Nucleus | -.45 | .23 |
| Total Caudate Nucleus | Right HVIIa Crus I | -.31 | .41 |
| Total HVIIa Crus I | Right Caudate Nucleus | -.47 | .21 |
| Right Caudate Nucleus | Total HVIIa Crus I | -.50 | .17 |
| Right HVIIa Crus I | Right Caudate Nucleus | -.47 | .20 |
| Right Caudate Nucleus | Right HVIIa Crus I | -.37 | .32 |

**Table S7:** Right and total caudate volumes of affected KE members have been previously shown to negatively correlate with complex non-word repetition scores [8]. Here we showed that their right and total HVIIa Crus I volumes also correlated negatively with these scores. We also showed that their HVIIa Crus I volumes positively correlated with their caudate nucleus volumes. In a series of partial correlations, we sought to examine whether right / total caudate volumes correlated with complex non-word repetition scores above and beyond right / total HVIIa Crus I volumes, and vice versa.
